# Anti-Neuroinflammatory Effects of Adaptogens: A Mini-Review

**DOI:** 10.3390/molecules29040866

**Published:** 2024-02-15

**Authors:** Dagmara Wróbel-Biedrawa, Irma Podolak

**Affiliations:** Department of Pharmacognosy, Jagiellonian University Collegium Medicum, Medyczna 9, 30-688 Cracow, Poland; dagmara.wrobel-biedrawa@uj.edu.pl

**Keywords:** adaptogen, neuroinflammation, pharmacological effects, *Schisandra chinensis*, *Eleutherococcus senticosus*, *Rhodiola rosea*, *Withania somnifera*

## Abstract

**Introduction**: Adaptogens are a group of plants that exhibit complex, nonspecific effects on the human body, increasing its ability to adapt, develop resilience, and survive in stress conditions. They are found in many traditional medicinal systems and play a key role in restoring the body’s strength and stamina. Research in recent years has attempted to elucidate the mechanisms behind their pharmacological effects, but it appears that these effects are difficult to define precisely and involve multiple molecular pathways. **Neuroinflammation**: In recent years, chronic inflammation has been recognized as one of the common features of many central nervous system disorders (dementia and other neurodegenerative diseases, depression, anxiety, ischemic stroke, and infections). Because of the specific nature of the brain, this process is called neuroinflammation, and its suppression can result in an improvement of patients’ condition and may promote their recovery. **Adaptogens as anti-inflammatory agents**: As has been discovered, adaptogens display anti-inflammatory effects, which suggests that their application may be broader than previously thought. They regulate gene expression of anti- and proinflammatory cytokines (prostaglandins, leukotriens) and can modulate signaling pathways (e.g., NF-κB). **Aim**: This mini-review aims to present the anti-neuroinflammatory potential of the most important plants classified as adaptogens: *Schisandra chinensis*, *Eleutherococcus senticosus*, *Rhodiola rosea* and *Withania somnifera*.

## 1. Introduction

Adaptogens are plants known for restoring the body’s endurance and strength, helping adapt to stressful conditions, and increasing mental and physical performance [1]. At the molecular level, these plant substances have a regulatory effect on cellular metabolism, resulting in the ability to produce a swifter and simpler stress response, thereby maintaining homeostasis [2,3]. They are regarded as having complex effects on the human body, including effects on the nervous, immune, and endocrine systems. Their unique and pronounced effects have led humans to use them for therapeutic purposes and as preventive remedies or body enhancers [4]. In many traditional healing systems, they have been used for hundreds of years as therapeutics, most often as tonic agents, restoring the correct balance in the body, providing support in conditions of abnormal stress, or even potentially prolonging life [1,4]. In addition, adaptogens have been reported to improve association and memory or even act as antidepressants and anti-anxiety agents. Not all the uses recommended by traditional medicinal systems have been confirmed and justified by current research, yet some results indicate that the activity profile of adaptogens may be broader than previously thought. Nevertheless, regardless of whether an effect is direct or indirect, their impact on the central nervous system seems unquestionable. Current uses of adaptogens include mainly conditions of stress and reduced physical performance as well as diseases where stress is an important factor underlying their pathogenesis (diabetes, cardiovascular diseases, anxiety disorders). Adaptogens are also considered of value in diseases that are accompanied by cognitive disorders [3].

At the body level, the mode of action of adaptogens is sometimes referred to as mild stress mimetics or stress vaccines, as the mild stress conditions they induce teach the body to withstand stress. This can be observed by a lower corticosterone output under experimentally induced stress conditions in subjects who received an adaptogen compared to controls who were not given this stress vaccine [5]. Furthermore, administration of these plant substances leads to various pharmacological effects, including anti-inflammatory activity. It has been reported that extracts of *Eleutherococcus senticosus*, *Rhodiola rosea*, *and Withania somnifera* decrease the expression of the key genes that govern the biosynthesis of pro-inflammatory leukotrienes in isolated brain cells [6]. Moreover, numerous studies have confirmed the anti-inflammatory potential of the best-known adaptogen, *Panax ginseng* (for review see: [7]). Its activity has been demonstrated both at the molecular level as well as in many in vivo models. As revealed so far, at the molecular level, the active compounds of adaptogens trigger antioxidant enzymes, neuropeptides (e.g., NPY), growth factors (e.g., BDNF), and signaling pathways that inhibit apoptosis [8]. They were shown to exert a protective effect on neurons and to stimulate neuro- or synaptogenesis. This mechanism may suggest a protective or even potentially therapeutic effect against CNS changes associated with ageing or neurodegenerative disorders (e.g., dementias), and psychiatric (e.g., depression, anxiety) or even oncogenic processes.

In recent years, it has been shown that one common feature of the abovementioned disorders is chronic inflammation that intensifies over time. The most common CNS diseases with accompanying inflammation are listed in Table 1.

With the discovery of the role of neuroinflammation in the development of many CNS disorders, the current search for potential therapeutics to combat these disorders has been extended to include substances with anti-inflammatory potential. A number of currently used medicinal substances have been tested in this regard. For example, the inhibitory effect on neuroinflammation accompanying spinal cord injury in rats under laboratory conditions has been demonstrated for the oral anti-diabetic drug, metformin [19], as well as for the lipid-lowering drug, ezetimibe, in rat middle cerebral artery occlusion [20]. Anti-inflammatory activity in animal models has also been reported for well-known SSRI and SNRI antidepressants (for Review—see [21]).

Importantly, many studies demonstrate that neuroinflammation can be mitigated by substances of natural origin, derived predominantly from plants. Numerous phytochemicals, analyzed so far, have been shown to exhibit anti-inflammatory properties with relatively low toxicity, and the mechanism behind this activity involves signaling pathways which are involved in the development of chronic inflammation within the CNS [22]. However, a key feature of the CNS-oriented effects, limiting the use of many plant-derived compounds, is the ability to penetrate the blood–brain barrier (BBB). Interestingly, plant-derived compounds with the potential to inhibit neuroinflammation may significantly differ structurally from each other. This may be attributed to the complexity of neuroinflammation and numerous sites of action where the vicious circle of chronic inflammation can be blocked. On the other hand, these numerous potential sites of action for natural compounds as well as their often low bioavailability make it very difficult to completely quench the inflammation process. A recently published review discussed phytochemicals that block inflammatory cellular responses associated with TNF-α signaling pathways [23]. Among the compounds listed in this paper were polyphenols (curcumin [24,25], 6-shogaol [26], epigallocatechin-3-gallate [27], resveratrol [28,29], luteolin [30], hesperetin [31]), terpenoids (e.g., ginsenoside Rg3 [32], tanshinone I [33]), and alkaloids (e.g., berberine [34]). Some of them (e.g., curcumin, resveratrol, epigallocatechin-3-gallate) have been included in clinical trials for neurodegenerative diseases (e.g., AD, PD, MS), indicating their high therapeutic potential.

This mini-review presents the results of recent studies, which demonstrate the anti-inflammatory potential of adaptogens with respect to the CNS while bearing in mind that treating the CNS as a separate system unaffected by what happens on the periphery is a false assumption. Nevertheless, certain compounds may exert a more potent effect on the brain, owing, for example, to their physicochemical properties and, consequently, bioavailability. Plants classified as adaptogens often contain unique groups of phytochemicals, which are responsible at least in part for their specific activity (e.g., schisandrins, withanolides). Thus, the assumption that these plant materials may be distinct in terms of activity, as well as use, is justified by their chemical composition. Furthermore, we aim to resolve if, despite the structural diversity of phytochemicals responsible for adaptogen activity, the mechanism leading to the suppression of neuroinflammation is to some extent common or whether generalizations are not possible. As the list of plant adaptogens is long, for the purpose of this mini-review, we have selected those most commonly used medicinally that already have European pharmacopeial monographs (*Schisandrae fructus*, *Eleutherococci radix*) or a European Medicine Agency (EMA) monograph (*Rhodiolae roseae rhizoma et radix*). *Withaniae somniferae radix* has also been included due to its enormous popularity in Europe which has been observed in recent years. Moreover, its EMA monograph is currently being prepared. On the other hand, due to the large amount of data on ginseng root and many reviews that have been published recently on various aspects of its activity and use, as well as the fact that a whole journal dedicated to *Panax ginseng* is available (Journal of Ginseng Research), this plant is excluded from the present mini-review.

## 2. Molecular Basis of Neuroinflammation

The physiological role of the inflammatory process is to combat any threats to the body. However, when inflammation becomes chronic, its protective role ends. Changes and reactions arising from an excessive inflammatory response and the consequent accumulation of pro-inflammatory factors, which spread throughout the body, may contribute to degenerative processes or disorders in the normal functioning of the organism’s machinery [35].

Inflammation in the central nervous system (CNS) can be initiated in response to infection, cell debris, trauma, or abnormal proteins, to give the best-known examples. Neuroinflammation differs from inflammation in other parts of the body in several fundamental aspects, one of which is, for example, the type of cells orchestrating the stages of initiation, development, and propagation of inflammation [36,37]. Indeed, these cells belong to microglia and astrocytes, which are unique to the CNS not only in terms of their location but also in the way they communicate with each other [38].

Microglia are specialized cells derived from macrophages found exclusively in the central nervous system. Given their origin, it seems clear that one of their roles is to perform an immune response in the specific environment of the CNS. They are also involved in the proper development of the nervous system, synaptic organization, neurotransmission, and protection and repair mechanisms when damage occurs. Many CNS diseases are characterized by microglia activation with accompanying inflammation. It is worth noting that in the case of neural tissue damage, activated microglia cells may polarize into M1 and M2 phenotypes to maintain homeostasis. The former is responsible for the pro-inflammatory response, while the latter acts to maintain balance by managing anti-inflammatory effectors. Astrocytes, which constitute the largest population of glial cells, control neuronal metabolism, which includes, for example, mediation between neurons and blood vessels. This determines their involvement not only in propagation but also in triggering inflammatory responses. Another cell type, oligodendrocytes, may also be involved in various stages of neuroinflammation. They may, for example, initiate a process associated with an autoimmune response targeting myelin [38].

In general, cytokines, chemokines, reactive oxygen species, and secondary messengers are involved in mediating the inflammatory process [39]. The networks between them are complex and link inflammation to further disorders. As such chronic inflammation within the CNS is an extremely complicated issue, only the most important factors or signaling pathways that contribute to this process will be briefly discussed below.

Considering sterile inflammation (unrelated to pathogen invasion), the intercellular concentration of ATP and glutamate increases in response to damage (neuronal death, metabolic disturbances due to lack of oxygen and glucose). Increased ATP levels stimulate purine receptors located on neurons, microglia, and astrocytes and lead to the formation of cytosolic inflammasome complexes, a key step in the induction of inflammation. NLRP1/NLRP2 inflammasomes are activated in neurons and astrocytes, while NLRP3 is activated in microglia [38,40].

Although there are differences between the effects of different inflammasomes, the overall outcome of their activation involves the recruitment of inactive pro-caspase-1 to form active caspase-1. Its main action is to cleave precursor pro-cytokines to produce active pro-inflammatory derivatives: interleukin (IL)-1β and IL-18. Caspase-1 can also induce inflammatory cell death, known as pyroptosis. Other cytokines released by activated microglia include tumor necrosis factor-α (TNF-α), prostaglandin E_2_ (PGE_2_), interferon-γ (IFN-γ), and other ILs, such as IL-6, IL-10, IL-23. These take part in the spread of inflammation along with interleukins; however, such factors as increased extracellular ATP, glutamate, and nitric oxide (NO) via Ca^2+^ waves initiated by astrocytes also contribute to this process. Such Ca^2+^ waves can propagate between astrocytes to regions located relatively far away in the brain. As microglia are sensitive to this signal, they can be recruited to migrate to the site of inflammation. NO synthesis is regulated by NO synthase (NOS) activity in microglia and astrocytes [38].

One of the most important factors in the recognition of harmful stimuli and the initiation of the inflammatory response are toll-like receptors (TLRs), which belong to pattern recognition receptors (PRRs). Several types of TLRs are involved in recognizing different injuries to the system, which can be endogenous (misfolded proteins, e.g., α-synuclein) or exogenous (e.g., lipopolysaccharide (LPS) from Gram-negative bacteria). Stimulation of TLRs leads to the activation of the nuclear factor kappa-light-chain-enhancer of activated B cells (NF-κB) pathway, which is essential for inflammasome formation. TNF-α is one of the key factors in inflammatory degeneration and cell death. It stimulates TRADD and TRAF2, leading to the activation of the NF-κB pathway, as well as c-Jun N-terminal kinase (JNK). The latter is involved in modulating apoptosis and inflammatory processes [36,38]. Janus kinase/signal transducer and activator of transcription (JAK/STAT) pathway should also be mentioned as a regulator of the inflammatory response modulating, for example, cytokine level [39].

Another pro-inflammatory signal transduction cascade involves mitogen-activated protein kinase (MAPK) pathways, which can be stimulated by the IL receptor. As a result, the production of IL-6 and IL-8 is observed. In addition, glycogen synthase kinase-3 (GSK-3) is recognized as a regulatory factor in inflammation. Inflammasome activation, recruitment of microglia to migrate, the impact on TNF-α, IL-6 and NO production in microglia, or increased BBB permeability are some examples of effects mediated by GSK-3 [40].

It is well known that eicosanoid production is dependent on cyclooxygenase (COX). Of the two isoforms of this enzyme (COX-1 and COX-2), which contribute to neuroinflammation, COX-2 seems to be the predominant form in the CNS and has become a major target in the search for anti-prostaglandin drugs in the context of neuroinflammatory disorders in recent years [41].

It is worth noting that, as a feedback loop to a highly inflammatory environment, anti-inflammatory pathways are simultaneously stimulated with released inflammation-inhibiting mediators. Among such examples is the phosphatidylinositol-3 kinase/protein kinase B (PI3K/AKT) pathway, which inhibits the proliferation of activated microglia, or the peroxisome proliferator-activated receptor γ (PPAR-γ), which suppresses the pro-inflammatory response triggered by activated microglia. PI3K/AKT and MAPK are involved in the nuclear factor erythroid 2-related factor 2 (Nrf-2). Its activation together with hemoxygenase-1 (HO-1) acts as an endogenous antioxidant regulator and can suppress inflammation [42].

**Figure 1:**
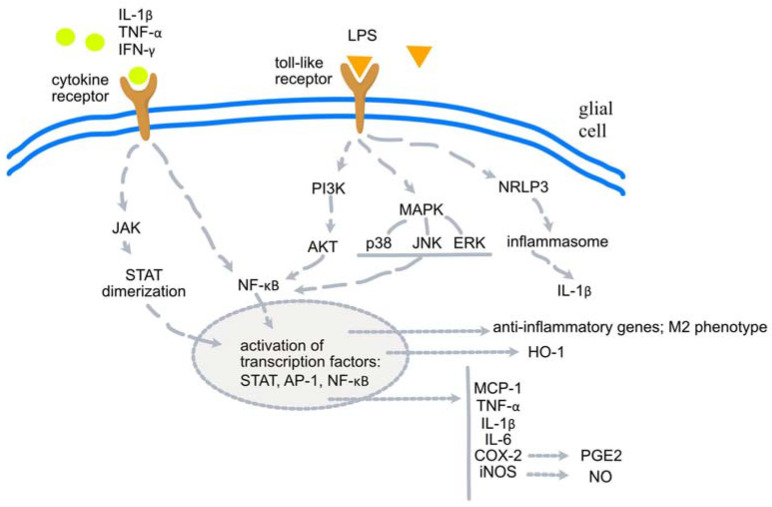
Cellular signaling pathways involved in the pathogenesis of neuroinflammation. AKT: protein kinase B; AP-1: activator protein-1; COX-2: cyclooxygenase; ERK: extracellular signal-regulated kinase; HO-1: hemoxygenase-1; IL: interleukin; iNOS: inducible NO synthase; JAK: janus kinase; JNK c-Jun: N-terminal kinase; M2: anti-inflammatory microglial phenotype; MCP-1: monocyte chemoattractant protein-1; NO: nitric oxide; NLRP: nucleotide-binding oligomerization domain, leucine-rich repeat, and pyrin domain; PI3K: phosphatidylinositol-3 kinase; STAT: signal transducer and activator of transcription.

The main pathways involved in the changes leading to neuroinflammation are shown in Figure 1.

Under physiological conditions, these pro-inflammatory/anti-inflammatory responses help maintain homeostasis. Nevertheless, when persistent inflammation cannot be cut off, the system is unable to maintain balance. An example can be neuronal death caused by β-amyloid accumulation, triggering an inflammatory reaction that is aggravated instead of being diminished by regulatory mechanisms. Similarly, neurotrophic factors (brain-derived neurotrophic factor, BDNF, glial-derived neurotrophic factor, GDNF, transforming growth factor β, TGF-β, vascular endothelial growth factor, VEGF, etc.) play a neuroprotective role and contribute to neuronal growth as well as synaptogenesis [38]. Under conditions of chronic inflammation, they also become immune modulators. Astrocyte-derived BDNF has been shown to attenuate immune reactivity in the glial environment, but TGF-β1, for example, can stimulate the spread of inflammation and impair neuroprotective mechanisms [38].

At the cellular level, chronic activation of glial cells can be observed during chronic nervous system inflammation, as well as inflammatory infiltration of the peripheral blood, myelin degradation, oligodendrocyte apoptosis, or axonal damage. Persistent activation of glial cells over a prolonged time, which occurs in chronic inflammation of the nervous system when homeostasis cannot be maintained, leads to excessive release of cytokines and other factors, some of which are neurotoxic (e.g., glutamate). This results in neurodegeneration and the maintenance of inflammation as a feedback loop. The most common neurodegenerative changes that affect most people are simply those that result from aging. Nevertheless, neuroinflammation also accompanies many psychiatric diseases (Table 1).

Furthermore, according to recent studies, neuroinflammation can also be triggered by peripheral disorders, such as chronic low-grade inflammation with metabolic dysfunction, which is described as meta-inflammation, or persistent dysbiosis of the gut microflora, which affects the immune system [43]. Activated immune cells (e.g., leukocytes) and cytokines (e.g., TNF, IL) can enter the CNS through the blood–brain barrier, which becomes permeable to peripheral inflammatory factors (cytokines have the effect of lowering the integrity of BBB components). It has been observed, for example, that intestinal T cells can migrate to the brain and mediate inflammatory damage in that site.

As outlined above, neuroinflammation is a complex process, the initiation and course of which is influenced by many factors. The data presented below refer to in vitro and in vivo studies performed for substances derived from selected plant adaptogens (whole powdered raw material, extracts, fractions, single compounds). It should be noted that experiments conducted on isolated cells or in animal models constructed to mimic specific disorders can only demonstrate a fraction of the reactions that may be taking place in the human body. Nonetheless, given the activities demonstrated to date, these results add to the current knowledge regarding the therapeutic potential of frequently used adaptogens.

## 3. Anti-Neuroinflammatory Activity of the Selected Adaptogens‘

### 3.1. Schisandra chinensis (Turcz.) Baill.

**Figure 2:**
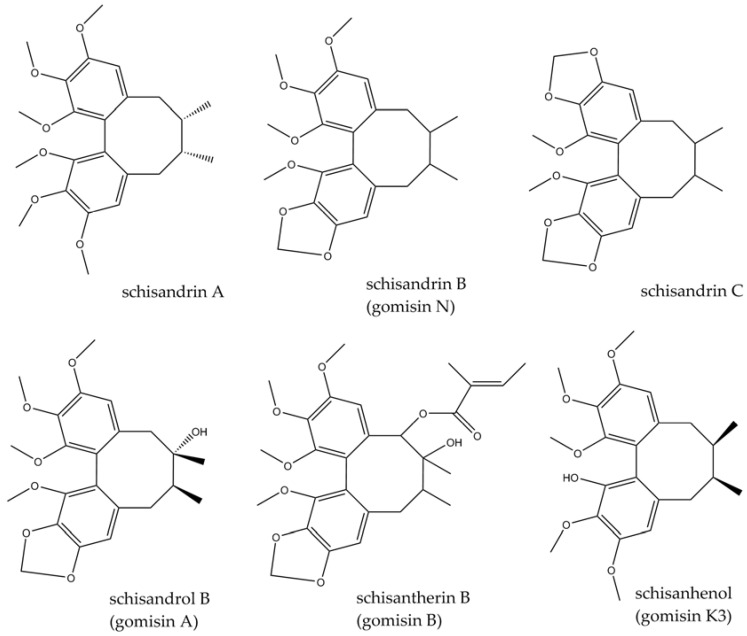
Structures of *Schisandra chinensis* lignans.

The species (syn. *Maximoviczia chinensis*) belongs to the Schisandraceae family and is a dioecious, climbing, perennial plant (liana) reaching from 0.5 to 25 m, found mainly in the eastern parts of Asia: China, Japan, and some regions of Korea, as well as Russia [5,44]. Its common names, such as Maximowich’s red grape, or Limonnik in Russian, refer to a characteristic lemon scent released by some parts of the plant. Other well-known vernacular names are *wu-wei-zi* (five-taste fruit) in Chinese and *gomishi* in Japanese [5]. The fruit (berries) have been used for medicinal purposes since ancient times for their tonic and sedative effects. The five tastes detected after their consumption (sour, salty, sweet, bitter, and astringent) have always attracted interest and aroused the curiosity of researchers. In traditional Chinese medicine (TCM), the fruit has been widely used to treat sexual disorders, urinary or gastrointestinal disorders, sweating, or body weakness [5]. In traditional Russian medicine, it has mainly been used to reduce weakness and improve vision, strength, and endurance. Schizandra fruit is a source of lignans, polysaccharides, essential oils, organic acids, and vitamins. Pharmacologically and structurally, lignans of the dibenzocyclooctane type appear to be the most interesting and attractive in terms of research. To date, according to [45], more than 40 different structures have been isolated, e.g., schisandrin A (deoxyschisandrin), schisandrin (schisandrol A), schisandrin B (γ-schisandrin, gomisin N), schisandrin C, schisanhenol (gomisin K3), schisandrol B (gomisin A), schisantherin A (gomisin C), and schisantherin B (gomisin B). Some of the structures are presented in Figure 2. Several properties, such as anti-inflammatory, antioxidant, anticancer, or neuroprotective, have been attributed to these unique compounds. Recently published review articles have discussed the potential use of Schisandra fruit and its active components as neuroprotective agents or an alternative therapeutical option in the treatment of neurological diseases [46,47,48,49]. In this review, we focus on mechanistic aspects concerning neuroinflammation and update the information with the most current publications.

In recent years, many studies have confirmed the anti-inflammatory properties of *Schisandrae* fruit, as well as its constituents that contribute to the biological efficacy of this plant material. Some of the results relate to CNS effects. *Schisandra* lignans (a total lignan fraction) were shown to inhibit activated MAPK inflammatory pathways in primary mouse neuronal cells incubated with Aβ1-42, which mimic Alzheimer’s disease conditions at the molecular level. The lignan fraction inhibited Aβ1-42-induced phosphorylation of JNK and p38, which is responsible for MAPK signaling [50]. One component of the lignan fraction, schisandrin C, has been shown to exert anti-inflammatory effects on mouse BV-2 microglia cells, reducing levels of TNF-α, IL-1β, IL-6, PGE_2_, and COX-2 expression [51]. It is known that the release of these cytokines is regulated by NF-κB and activator protein-1 (AP-1). Schisandrin C inhibited their nuclear translocation and stimulation of gene expression for inflammatory mediators. Furthermore, MAPK (e.g., p38, ERK, JNK) pathways, which activate NF-κB, and janus kinase/signal transducer and activator of transcription (JAK/STAT) were also inhibited. The anti-inflammatory effect of this lignan was also linked to the activation of Nrf-2 and the cAMP/PKA/CREB pathways. The expression of iNOS was also inhibited in this study. These results are similar to those reported earlier by Joo et al. [52], who showed a reduction in IL-1β, NO activity, and inhibition of NF-κB nuclear translocation caused by dimethyl biphenyl dicarboxylate, a synthetic schisandrin C derivative. These results were later repeated for schisandrin C itself as well as for schisandrin A. In the latter case, a reduction in LPS-induced increases in TNF-α, IL-6, and NO levels was demonstrated in mouse primary microglia and BV-2 cells [53]. This was attributed to the suppression of NK-κB and JAK2/STAT2, as well as to STAT3 nuclear translocation. Decreased levels of TNF-α, IL-1β, IL-6, and PGE_2_ were observed in BV-2 cells along with the inhibition of the LPS-activated NK-κB pathway for schisandrin B (gomisin N) [54]. The authors found that these changes may result from PPAR-γ activation. In another study, the downregulation of mRNA expression levels for TNF-α, IL-1β, IL-6, PGE_2_, COX-2, or iNOS in LPS-stimulated mouse BV-2 cells was also demonstrated [55]. Furthermore, using a model of depression induced by LPS administration to mice, the antidepressant effect was demonstrated by the same team in a forced swim test of gomisin N (100 mg/kg *p.o*.), accompanied by an attenuation of inflammation simultaneously induced by LPS administration [55]. Inhibition of inflammation-related gene expression in the hypothalamus and amygdala was observed as well. Similar effects have been reported in vivo for schisandrin B by other authors in rat models of brain injury [56,57]. Furthermore, given the anti-inflammatory potential of schisandrin B, Lam et al. [58] tested the effect of its combination with the antiparasitic drug, albendazole, in *Angiostrongylus cantonensis*-induced meningoencephalitis in BALB/c mice. The administration of 20 mg/kg/day of schisandrin B for 7 days (third week after infection) following weekly administration of albendazole (same dosage) *p.o.* resulted in the inhibition of inflammasome formation (inhibition of transcription factors for inflammasome components such as NLRP1B and NRLC4), and mediators arising from inflammasome activation (caspase-1, IL-1β, IL-18) were also shown to be reduced.

Moreover, pyroptosis in the murine brain tissue was reduced. The inhibition of NLRP1-inflammasome-induced neuronal pyroptosis (and neuronal apoptosis) induced by the presence of Aβ deposits in AD-induced transgenic mice was the result of a 2-week administration of 2 mg/kg/d schisandrin (the exact lignan was not indicated) [59]. This was accompanied by a downregulation of gene expression in brain tissue for caspase-1, IL-1β, and IL-18. Concurrently, a reversal of cognitive impairment, assessed by the Morris water maze test, was observed at the behavioral level. An anti-inflammatory effect in rodents with induced AD was also demonstrated by Song et al. [60] for schisandrin (again, it was not stated which lignan exactly was used). In this study, the investigators used Wistar rats with dementia induced by streptozotocin administration. Here, too, they demonstrated a reduction in TNF-α, IL-1β, and IL-6 levels, and the inhibition of NK-κB pathway activation in brain tissue collected from animals that received 2 or 4 mg/kg/day of schisandrin *i.p.* for 2 weeks. At the same time, the study group showed a reversal of the cognitive impairment characteristic of animals with model AD (weaker effect for the 2 mg/kg/day dose), assessed by the same test as before. In a recent paper [61], the authors examined if the antidepressant effect of *Schisandra* lignans (composition not specified) in mice with model depressive symptoms induced by chronic unpredictable mild stress is related to the knockdown effect of neuroinflammation. Improvements in depressive-like symptoms were demonstrated for the lignan mixture administered at a dose of 600 or 1200 mg/kg by gastric gavage in a forced swim test, while dose dependency was observed, and the effect for the higher dose was comparable to that of fluoxetine administered at 10 mg/kg *i.p.* At the same time, a reduction in protein expression of inflammatory mediators (TNF-α, IL-1β, IL-6, COX-2, iNOS) was demonstrated in hippocampal tissue collected from the animals. Furthermore, the inhibition of the JAK2/STAT3 signaling pathway, the activation of which must occur in mediating neuroinflammation by microglia, was demonstrated. In vitro analysis in BV-2 microglia cells showed that lignans have the ability to polarize microglia cells towards promoting the M2 phenotype, which is responsible for the anti-inflammatory component of microglia activity. This process occurs through the inhibition of the JAK2/STAT3 pathway and activation of STAT6. As cannabinoid receptor 2 (CB_2_R) is also involved in promoting the differentiation of microglia cells towards M2, the effect of lignans on this molecular target was further examined. Indeed, it appeared that the stimulation of the CB_2_R/STAT6 pathway was responsible for the beneficial anti-inflammatory effect.

Gomisin A exhibited effects that are consistent with those obtained for the schisandrins. Gomisin A reduced levels of TNF-α, IL-1β, and IL-6 in N9 microglia cells through the inhibition of the inflammatory mediator-inducing effect of NF-κB [62]. In addition, the inhibition of the MAPK pathway was observed, as well as the protein expression of TLR4, a key protein in stimulating the aforementioned signaling pathways of inflammation.

Yet another group of compounds found in the *Schisandra* fruit that have been claimed to have neuroinflammation-lowering activity are polysaccharides. *Schisandra* crude polysaccharide (SCP) and its purified fraction, SCP-2, are composed of glucose and galactose. Xu et al. [63] showed that SCP-2 (56 mg/kg/day for 3 weeks, with LPS administered for the last 9 days) abolished memory impairment induced by LPS-induced neuroinflammation in mice. The effect was related to the reduction of pro-inflammatory cytokines, with a significant effect on TNF-α, the restoration of microglia activation to basal levels that were stimulated by LPS administration, and the reduction of NF-κB or MAPK pathway activity. Similarly, a more recent paper from 2023 [64] presented a plasma-lowering effect of pro-inflammatory cytokines in rats with Aβ_25-35_-induced dementia for both SCP and SCP-2 (20 mg/kg/d SCP administered orally for 4 weeks, followed by 50 mg/kg/d SCP or SCP-2 for another 4 weeks). In addition, both polysaccharide fractions inhibited microglia activation along with restoring normal neuronal morphological structure and number. An additional effect was the restoration of a favorable composition of the gut microbiota, disruption of which can also induce adverse inflammatory changes in the CNS. In addition, the essential oil isolated from the stems of the plant was also tested [65]. Apart from certain memory-enhancing effects (dosage: 67 or 200 mg/kg/day, 2 weeks) in SD rats with Aβ_1-42_-induced dementia, which was, however, weaker than that of the reference compound, donepezil (dosage: 20 mg/kg/day, 2 weeks,), significant reductions in pro-inflammatory cytokines (TNF-α, IL-1β) and neurotrophic factors (GDNF, BDNF, NGF) were demonstrated. A higher dose of essential oil was more effective. Furthermore, a reduction of inflammatory mediators’ expression in the striatum of mice with model Huntington’s disease induced via the administration of 3-nitropropionic acid (3-NPA) was shown for micrandilactone C, a schiartane nortriterpenoid, recently isolated from *S. chinensis* roots (doses of 1.25 and 2.5 mg/kg/day i.v., 5 days) [66]. Downregulation of the STAT3 signaling pathway was also observed, as well as the inhibition of microglia activation induced by 3-NPA administration.

### 3.2. Eleutherococcus senticosus (Rupr. & Maxim.) Maxim.

**Figure 3:**
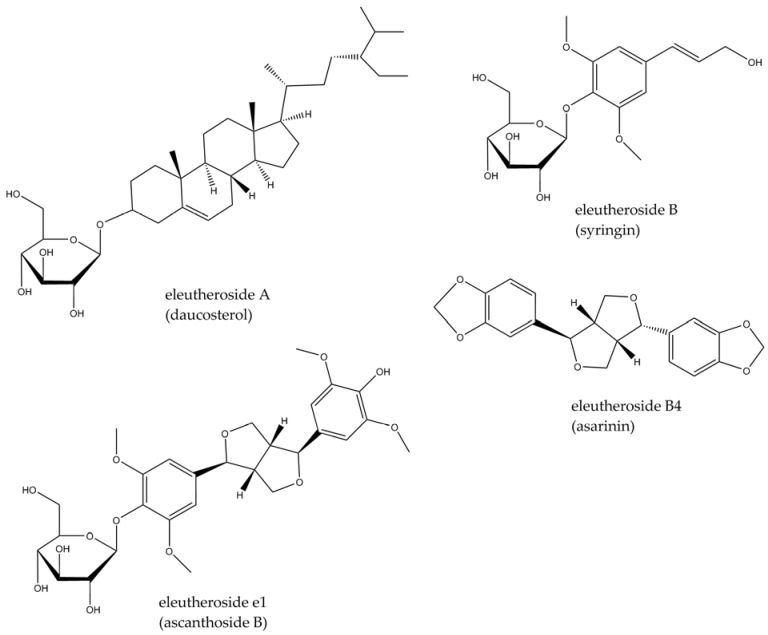
Structures of eleutherosides found in *E. senticosus*.

The species (syn. *Acanthopanax senticosus*) belongs to the Araliaceae family and is a woody shrub, usually 2, but up to even 7 m long, with erected stem, thorny and branched sparingly. The leaves are three–five foliate, prickly or smooth, and the fruits are berries, black when mature [67,68]. The most widely known vernacular names are Siberian ginseng, dyavol’skii kust (devil’s bush), or nedotroga (untouchable) [67]. Despite its name (Siberia is a region in northern Russia), it is widely distributed in eastern Asia and used not only in the Russian traditional medicinal system but also in others. In Ayurveda, it is treated as a rasayana—a rejuvenating medicine—while in Malaysian, Indonesian, and Chinese medicine it plays the role of a tonic and regenerative agent. The rhizomes or roots are usually used for medicinal purposes, but other parts of the plant, such as leaves, stems (as well as stem bark), and fruit, are also popular [69]. The active constituents of *Eleutherococcus senticosus* include lignans, triterpene saponins, and phenylpropanoid derivatives called ciwujianosides or eleutherosides (with different letters added depending on the structure), found mainly in the roots (Figure 3). In addition to its medicinal use, the plant is a well-known and widely consumed functional food ingredient. A review outlining its therapeutic effects and applications in CNS disorders was recently published [70], showing that a partly anti-inflammatory activity is important in its clinical effect.

There are several studies indicating the anti-inflammatory and immunomodulatory activity of extracts or isolated active constituents of this plant. For example, dried methanolic extract from stem bark and roots has been tested for its effects on macrophage polarization and plasma cytokine levels [71], whereas an intract obtained from the fruits has been studied to examine its effects on plasma leukocyte levels [72]. As regards isolated phytoconstituents, eleutheroside B (Figure 3) has been reported to have an inhibitory effect on COX-2 (in silico study) [73]. However, there are relatively few papers which have focused on *E. senticosus* efficacy effects in the CNS context. Those published in recent years are presented below.

In an in vitro study on glial-derived T98G cells, extracts of *E. senticosus, W. somnifera*, and *R. rosea* were shown to inhibit the expression of genes involved in leukotriene synthesis, which inhibits the leukotriene signaling pathway of neuroinflammation and may confer beneficial effects in conditions such as AD [6].

A 70% ethanolic extract of *Eleutherococcus* stem bark showed anti-neuroinflammatory activity as assessed through gene expression for COX-2 and GFAP in rats with induced global cerebral ischemia [74]. The drug was administered orally, and its effect was dose-dependent at 300 mg/kg given twice after reperfusion. In the Wang et al. [75] study on rats, several enzymes, key to the beneficial effects of *E. senticosus* leaf extract in the treatment of ischemic stroke, were selected. These included COX-2 and iNOS.

In view of recent scientific reports regarding the role of the gut–brain microbiota axis in the development and treatment of inflammatory CNS disorders in determining the mechanism of action of *Eleutherococcus* leaf extract in the treatment of ischemic stroke its effect on gut bacterial composition was also considered [76]. Oral administration of the plant material seems therefore to be crucial, as is the case in traditional medicinal systems of the Far East. An aqueous solution of freeze-dried ethanolic extract of dried *E. senticosus* leaves was administered at a dose of 100 mg/kg/day for 4 weeks to SD rats after ischemic stroke. Results obtained suggested that the extract was able to restore the imbalance in the composition of the intestinal microflora, as well as lower the levels of pro-inflammatory cytokines (TNF-α, IL-1β, IL-6) and increase the levels of anti-inflammatory IL-10, both in brain tissue and plasma. The regulating effect of inflammatory cytokines (TNF-α, IL-6, IL-10) in neuroinflammation accompanying induced ischemic stroke in rats was, moreover, demonstrated in an earlier study [75].

A study by Huang et al. [77], in turn, showed positive effects, resulting from the administration of the saponin fraction from *E. senticosus* leaves, on learning and memory functions in mice, with additional modulatory effects on signaling pathways that play an important role in neuroinflammation: PI3K/AKT and MAPKs.

### 3.3. Rhodiola rosea *L.*

**Figure 4:**
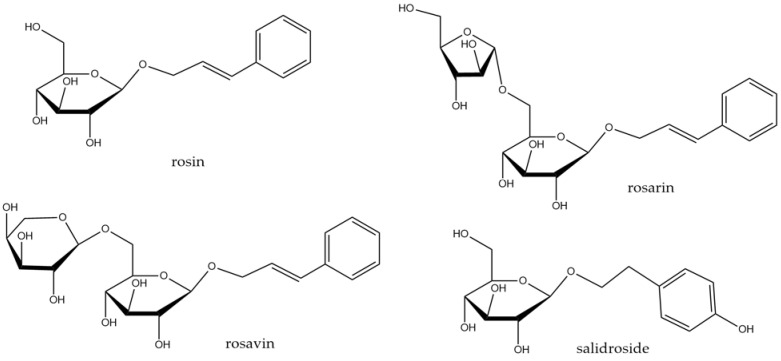
Structures of compounds found in *R. rosea*.

The species belongs to the Crassulaceae family. It is a perennial plant, reaching up to 70 cm, with yellow blossom and thick rhizome, fragrant when cut, widespread in the mountainous regions of Asia, Europe, and North America [44]. It is commonly known as golden root, roseroot, or arctic root. It is an important ingredient in the traditional medicine of eastern Europe and Asia. Roseroot is usually used to increase physical performance and strength, and to eliminate fatigue or symptoms of depression. The most characteristic groups of compounds in the root are phenylpropanoids (rosavin, rosin, and rosarin shown in Figure 4) and phenylethanol derivatives (salidroside, Figure 4). Other constituents include flavonoids, pronanthocyanidins, phenolic acids, or triterpenes. The essential oil with geraniol and its derivatives is responsible for the characteristic rose fragrance. Several reviews on *R. rosea* and its constituents with potential evidence-based therapeutic use have recently been published [78,79].

In LPS-stimulated mouse BV-2 microglial cells, crude *R. rosea* extract (methanolic fraction of aqueous extract), rosin, rosarin, and salidroside (Figure 4) reduced gene expression for iNOS and cytokines (TNF-α, IL-1β, IL-6) [80]. The same effect was observed peripherally in murine brains after a single oral dose of 500 mg/kg of the extract in LPS-injected mice. MAPK pathways (JNK, p38) appear to be involved in the activity of these components. Ethanolic extract of *R. rosea* roots and rhizomes attenuated neuroinflammatory response in corticotropin-releasing hormone (CRH)-stimulated BV-2 cells that were a model of stress conditions [81]. The extract reduced IL-6 levels, inhibited NF-κB nuclear translocation, and inhibited MAPK phosphorylation and activation: MAPK-activated protein kinase 2 (MMK2), ERK ½, and JNK.

Furthermore, in a murine model of autoimmune encephalomyelitis, *R. rosea* (standardized for salidroside content and administered intragastrically; the amount of the plant was calculated to contain a dose of 100 mg/kg/day of salidroside) showed a regulatory effect on JAK1, JAK2, and STAT3 pathways in the spinal cord, with peripheral modulation of cytokine levels (decrease in IL-6, IL-17A, IFN-γ; increase in IL-4) [82]. In a meta-analysis of publications on *R. rosea* efficacy in ischemic stroke, preclinical data proved its ameliorating effect on neuroinflammation (significant decrease in TNF-α) [83]. Furthermore, salidroside was demonstrated to reduce glial activation in LPS-induced brain neuroinflammation in mice (caspase-3 and GFAP gene expression) [84]; it was also active in a model of transient middle cerebral artery occlusion in rats via the PI3K/PKB/Nrf-2/NF-κB signaling pathway [85]. Similar results were obtained by Xu et al. [86], who proved the involvement of the Nrf-2/HO-1/NF-κB pathway in reducing inflammation in LPS-induced cognitive impairment in rats.

### 3.4. Withania somnifera Dunal

**Figure 5:**
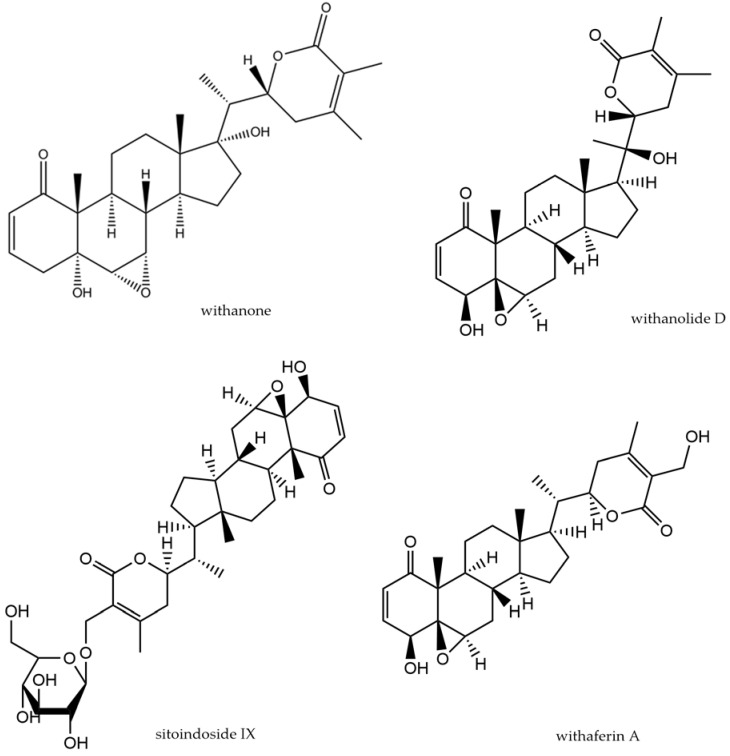
Structure of compounds found in *W. somnifera*.

The species belongs to the Solanaceae family and is a shrubby plant distributed especially in India, Africa, southern Europe, and Australia [44]. It is commonly known as ashwagandha or Indian ginseng. Its root has been widely used in Ayurvedic medicine as rasayana, meaning a rejuvenation drug. Its applications have covered states of exhaustion, including lack of energy in the elderly, immune weakness, and osteo-muscular disorders. The chemical composition of ashwagandha is extremely interesting and varied. The main active compounds are steroidal lactones (withanolides and withaferins, shown in Figure 5) and their glucosylated derivatives (sitoindoside IX—Figure 5—and X), steroidal saponins with an additional acyl group (sitoindoside VII and VIII), and alkaloids (e.g., isopelletierine, which occurs also in other plants). Several recent review publications focused on the use of ashwagandha in CNS disorders and collected data on the pre- and clinical activity of powdered raw materials, extracts, and individual active ingredients (e.g., [87,88]).

200 mg/kg/day of ethanolic extract of *Withania somnifera* roots administered daily to rats for 30 days, one hour before AlCl3 (a neurotoxic agent), showed a protective effect demonstrated by a decrease in the levels of parameters indicative of brain tissue damage [89]. Attenuation of TNF-α levels in the hippocampus and cortex collected from the animals was observed.

Withanone (Figure 4), isolated from *Withania* roots, administered orally for 21 days (doses: 5, 10, 20 mg/kg/day) alleviated neuroinflammation associated with cognitive impairment in streptozotocin-induced dementia in rats [90]. Levels of TNF-α, IL-1β, IL-6, and monocyte chemoattractant protein-1 (MCP-1) in brain tissue homogenates were significantly reduced. In addition, a reduction in peripheral pro-inflammatory cytokines (IFN-γ, TGF-β, IL-2, IL-17) was also observed, but no increase in anti-inflammatory factors (IL-4, IL-10) was detected. The cognitive performance of the rodents, tested in a passive avoidance test, was simultaneously significantly improved by the two higher doses of withanone.

Another important component of *Withania somnifera* is withanolide A. Its ability to attenuate glial activation and inflammation in the hippocampus induced by pilocarpine-induced status epilepticus in mice was demonstrated by Zhu et al. [91]. Withanolide A, administered orally at the dose of 5 mg/kg/day for 3 consecutive days after seizure arrest, reduced levels of TNF-α, IL-1β, and glial fibrillary acidic protein (GFAP). Furthermore, in another study, the compound was shown to reduce NF-κB gene expression, attenuating AD pathogenesis in mice, while decreasing NLRP3 and inflammasome activation [92]. As shown previously, NRLP3 and NF-κB activation leads to the attenuation of Aβ phagocytosis by microglia, which increases the number of Aβ deposits in brain tissue and intensifies inflammatory response [93].

A protective effect against memory impairment (tested by Y-maze test, NOR test, and modified elevated plus maze test) and neuroinflammation in mice with thioacetamice-induced hepatic encephalopathy were shown for *W. somnifera* root extract administered for 29 days (200 or 400 mg/kg) [94]. Protein expression for TNF-α, MAPK (p38, ERK1/2), and NF-κB was shown to be inhibited in both the peripheral (liver tissue) and brain tissue, with concomitant upregulation of Nrf2 and HO-1. The effect was more significant for the higher dose of the extract.

As has been demonstrated, drug abuse can cause cognitive and mental impairment accompanied by neuroinflammation. *W. somnifera* extract (100 mg/kg, i.p.) caused a reversal of (+/−)-3,4-methylendioxymethamphetamine (MDMA)-induced impairment in mice and suppressed microglial activation in the striatum and substantia nigra pars compacta in mouse brains. The results of this study also indicate reduced microglia activation in the substantia nigra and hippocampus after 5–6 weeks of treatment with *Withania r*oot extract (50% ethanolic in a rotenone-induced Parkinson’s disease model in rats) [95]. Unfortunately, the inconsistency of dosage presented in the text makes it difficult to draw clear conclusions from the research.

As an alternative to the use of the root, which requires the entire plant to be destroyed, research is being conducted to exploit other parts of the plant. It has been shown that the leaf can also be a source of bioactive steroidal lactones such as withaferin A or withanone. Its inhibitory effect on neuroinflammation has been tested in the context of anxiety disorders resulting from metabolic conditions [96]. In mice given a high-fat diet leading to obesity, when dried leaf powder (1 g/kg/day) was administered concomitantly with food, improvements in anxiety-related behavior (measured using the elevated plus maze test) were observed. Moreover, a reduction in the expression of proteins in brain tissue indicative of glial fibrillary acidic protein (GFAP) activation, as well as inflammatory factors TNF-α, IL-1β, IL-6, iNOS, COX2, chemotactic factor MCP-1, and down-regulation of TLR4, together with a reduction in activation of the NF-κB pathway, were seen.

A similar mechanism of action of fractions containing the aforementioned steroidal lactones was proposed in an in vitro study on LPS-stimulated BV-2 microglia cells [97]. Other authors have also demonstrated the downregulation of NO levels and the upregulating effect of the Nrf2 pathway, which in turn resulted in the activation of heme oxygenase-1 (HO-1), showing immunomodulatory effects, while inhibiting NF-κB, in an in vitro mechanistic study on BV-2 cells [98]. Similar results of neuroinflammation quenching were observed for an aqueous extract of ashwagandha leaves (ASH-WEX), which simultaneously reduced LPS-induced systemic administration or sleep deprivation-induced anxiety behavior in rats [94] and, in another experiment, improved impaired cognitive function [99]. The mechanism of action was related to the inhibition of NF-κB and MAPKs (p38 and JNK). Since the extract was administered 8 weeks (140 mg dry extract/kg/day, orally) prior to LPS injection, the demonstrated effect can be considered protective against the development of neuroinflammation in the course of various diseases.

In a clinical trial involving 66 individuals with diagnosed schizophrenia or schizoaffective disorder, taking appropriate antipsychotic medication, who nevertheless experienced severe negative symptoms, an adjuvant in the form of an encapsulated standardized extract of *Withania somnifera* (Sensoril^®^, specified minimum content of withanolide glycosides, with trace content of withaferin A) was used [100]. The dosing regimen was as follows: week 1—2 × 250 mg extract; from week 2 onwards for further 11 weeks— 2 × 500 mg extract daily, with a placebo control (capsule with neutral content). It was shown that treatment with *W. somnifera* extract resulted in a significant alleviation of negative symptoms and an improvement of the general condition and symptoms related to the perception of stress but with no effect on the positive symptoms. Inflammatory parameters were also assessed, and a decrease in plasma CRP and S100B protein levels was detected, but this was not statistically significant compared to placebo. In contrast, there was virtually no change in IL-6 levels (while IFN-γ, IL-2, and IL-4 levels were undetectable).

As Ashwagandha extracts have been shown to reduce NF-κB activation, and this signaling protein is recognized as one of the key molecular targets for the treatment of transactive response DNA binding protein 43 (TDP-43)-related proteinopathies (including amyotrophic lateral sclerosis (ALS) and frontotemporal degeneration (FTLD)), the potential of the plant and its constituents to alleviate symptoms of these disorders has been verified [101,102]. The authors showed that Ashwagandha root extract in transgenic mice with ALS (5 mg per mouse, by gavage, every other day for 16 weeks) or withaferin A in FTLD transgenic mice (5 mg/kg, *i.p.,* every other day for 8 weeks) improved motor (ALS) and cognitive (ALS, FTLD) performance while reducing NF-κB activity and inflammation in the brain. In a study by Dutta et al. [101], levels of inflammatory cytokines in microglia (wild-type and TDP-43A315T) were checked in vitro. The extract reduced elevated levels of pro-inflammatory IFN-γ, IL-1β, IL-6, IL-17, and MCP-1, and increased levels of anti-inflammatory IL-4.

## 4. Discussion

### 4.1. Adaptogens in Neuroinflammation

Research from recent years shows that chronic inflammation (neuroinflammation) is a common component of many diseases of the central nervous system. Furthermore, inflammation accompanying peripheral conditions (e.g., metabolic syndrome) can spread and extend to the CNS, once regarded as separate from the rest of the body. When mechanisms designed to maintain homeostasis in the organism, rather than having a restorative function, take on pathogenic characteristics as a result of positive coupling, the inflammatory process may be sustained, leading to the subsequent development of neurological conditions.

The growing awareness of the role played by inflammatory processes in the pathogenesis and development of CNS disorders is drawing the attention of researchers and clinicians to the preventive and therapeutic potential of substances that mitigate inflammation, particularly neuroinflammation. Of particular interest within this group are substances of plant origin, and amongst these are the ones that possess psychotropic potential. Such plant materials include adaptogens. The results of studies on anti-inflammatory activity presented in this mini-review concern the four most commonly used adaptogens (*Schisandra chinensis*, *Eleutherococcus senticosus*, *Rhodiola rosea*, *and Withania somnifera*), excluding Panax ginseng root, to which a separate monograph should be devoted.

The results presented here confirm that these plants exert an inhibitory effect on neuroinflammation at both the initiation and propagation stages of inflammation (Table 2). Experimental data regarding this effect was derived from in vitro studies on isolated cell lines as well as in vivo models, usually in the context of specific CNS disorders experimentally induced in rodents. In the latter case, the levelling of neuroinflammation was usually accompanied by an improvement in behavioral symptoms indicative of CNS disorders, e.g., memory deficits, helplessness, elevated anxiety, and motor disorders. This may indicate both the preventive potential of the applied plant substances and the amelioration of symptoms of these disorders. Moreover, perhaps they are even able to break the vicious circle of positive feedback sustaining permanent inflammation.

### 4.2. Limitations of Studies

The use of traditional agents as mental tonic agents and the very large number of studies confirming the central effect of these substances prove that the active compounds (or presumably their metabolites) have the ability to penetrate the blood–brain barrier. Hence, the problem of bioavailability is eliminated, which concerns most compounds of natural origin and demands the preparation of a suitable formulation. Oral administration, which is the most desirable route for human use (ease of use and self-precise dosing), was the most common in the studies cited. Nevertheless, the effective doses were usually very high compared to the typically used reference drugs. They generally ranged from a few tens to as much as 500 mg per kg body weight, while, for example, doses of antidepressants usually used in behavioral studies fall in the range of 10–20 mg/kg, whereas effective doses of reference benzodiazepine anxiolytics are usually 5 mg/kg or less, and of anti-Alzheimer’s donepezil are most often up to 10 mg/kg. Most of the published experiments unfortunately lacked reference compounds, which on the one hand is quite surprising as this is the standard for scientific papers, but on the other hand, it is difficult to compare substances whose effects on the body are so complex with standard drugs.

The use of herbal remedies represents an alternative to Western medicine, which relies on isolated compounds focused on single molecular targets. Adaptogens, as part of traditional medicinal systems, are ingested orally in the form of preparations (e.g., decoctions), which necessitates the consumption of complex mixtures of compounds responsible for the complex effects these plant remedies exert on the organism. This justifies the use of extracts, as opposed to isolated phytochemicals, in most of the published studies. As already mentioned, it is very difficult to identify unequivocally one or two compounds that would be exclusively responsible for the action of these complex extracts. In most cases, it is in combination that the full effect of these raw materials can be achieved due to their synergistic action towards each other. Furthermore, in many countries, products containing adaptogens are sold as dietary supplements and not as herbal medicinal products. Dietary supplements are usually not standardized for the content of individual compounds or groups of active compounds, so assessing their quality is difficult.

However, there are also limitations in translating the experimental results into preventive or therapeutic applications. In many of the studies conducted, insufficient attention was paid to defining the chemical composition of the extracts used. The activity of extracts, which are mixtures of numerous compounds of different physico-chemical properties as well as individual modes of action, is extremely complex to assess. Some molecules may potentiate the effect of others, exert additional pharmacological properties that are beneficial in a particular case (e.g., in the case of antidementia, in addition to anti-neuroinflammatory effects, this may be acetylcholinesterase inhibition), or simply constitute ballast substances. The content and level of the respective compounds will therefore determine the effect as well as its reproducibility and, in some cases, also safety. Nevertheless, some extracts, especially those tested clinically, have been standardized. On the other hand, adaptogenic raw materials contain unique structures, not found in other plants, whose specific activity was little known until recently. Studies on isolates are therefore also very valuable, as they provide information on key components that contribute to the observed efficacy, and this should be considered in standardization.

### 4.3. Safety of Adaptogens

The use of the aforementioned adaptogens is also beneficial due to their fairly high safety profiles. These plants have been part of traditional medicinal systems for centuries and are therefore known, based on experience, to be non-toxic, and some are even classified as GRAS (generally recognized as safe). Their potential preventive use should therefore not be objectionable. Some can be added to functional foods, of course taking into account food standards and possible intervals of use.

As such, there are few studies that have examined the safety profile of adaptogens; nevertheless, those that have assessed this aspect have shown good tolerability by patients and only mild side effects. This was the case for *Rhodiola rosea* extract WS^®^ 1375 administered at a dose of 200 mg/kg, twice daily for 4 weeks [104]. Thirty-six per cent of patients experienced adverse reactions of minor severity—most commonly neurological (e.g., dizziness) or gastrointestinal symptoms. Similarly, *W. somnifera* produced only mild and mainly transient side effects that involved somnolence and gastrointestinal disorders, and, less commonly, drowsiness, vertigo, hallucinations, or nasal congestion [105]. In the case of *E. senticosus*, arterial hypertension was recognized as a contradiction for use [106]. In turn, *S. chinensis* in a study by Kim [107] did not induce any serious negative effects. Only painful complaints were recorded; however, no clear association with the extract was observed. However, it should be noted that all of these clinical observations described were conducted on small groups of patients.

## 5. Conclusions

Adaptogens seem to have fascinated therapists for centuries because of their unique effects, in particular rejuvenation. This effect, which is most often attributed to these plant substances, may be due to the inhibition of degenerative processes that progress in our body with age. In the case of many CNS disorders, these processes take on an aggravated and disorder-specific form. They are also accompanied by inflammatory changes. The knowledge of the mechanisms determining the pharmacological effect of adaptogens may therefore be a justification for extending their existing applications or designing studies to evaluate their efficacy in disorders in which these plant substances have not previously been used.

It seems that the presented data justifies further exploration of the adaptogens in CNS disorders with concomitant neuroinflammation or of inflammatory origin.

## 6. Materials and Methods

Pub-Med and Scopus databases were using keywords “neuroinflammation + Schisandra chinensis”, “neuroinflammation + Withania somnifera”, “neuroinflammation + Eleutherococcus senticosus”, and “neuroinflammation + Rhodiola rosea”. The scientific articles from the years 2013–2023, written in English, were included. When the effect assessed in the experiment concerned two plant materials administered together, the article was excluded.

## Figures and Tables

**Table 1:** Main CNS diseases and disorders with accompanying neuroinflammation.

CNS Disorder	Main Pathogenetic Factor	Ref.
Alzheimer’s disease (AD)	extracellular Aβ amyloid plaques and intracellular hyperphosphorylated τ-protein neurofibrillary tangles; cholinergic neuron loss; neurodegenerative	[9]
Parkinson’s disease (PD)	intracellular aggregates of α-synuclein; dopaminergic neuron loss in *substantia nigra pars compacta*; neurodegenerative	[10]
Huntington’s disease(HD)	aggregates of misfolded huntingtin; shrinkage of brain; neuron loss in *striatum*; neurodegenerative	[11]
Multiple sclerosis (MS)	auto-immune-mediated demyelination of neurons; neurodegenerative	[12]
Depression	dysregulated neuroplasticity monoamine neurotransmission; neuroendocrinal function	[13]
Anxiety disorders	disturbances of neurotransmittency	[14]
Schizophrenia	disturbances of neurotransmittency; altered connectivity and neuroplasticity in neurodevelopmental period	[15]
Ischemic stroke	occlusion of cerebral artery, e.g., by thrombus with subsequent regional decrease of oxygen to the brain;	[16]
Infections	bacteria (e.g., *Escherichia coli*, *Neisseria meningitidis*); viruses (e.g., *Ebola virus*, *HIV)*; protozoa and helminths (e.g., *Toxoplasma gondii*); fungi (e.g., *Cryptococcus neoformans*)	[17]
CNS cancer	gene alteration; oxidative damage; environmental causes (e.g., diet); infections	[18]

**Table 2:** Main anti-neuroinflammatory effects of substances derived from *S. chinensis*, *E. senticosus*, *R. rosea*, and *W. somnifera* found in studies (see text above and references for further details).

Observed Effect	Substance	Assay/Model	Ref.
↓TNF-α	schisandrin A schisandrin B (gomisin N) schisandrin C schisandrol B (gomisin A) *Schisandra* lignans SCP-2 *Schisandra* essential oil *E. senticosus* extract salidroside *R. rosea* extract, rosin, rosarin, salidroside *R. rosea* extract withanone withanolide A *W. somnifera* extract	BV-2 microglial cells, mouse primary microglia micorglia–neuron cocultures BV-2 microglial cells forced swim test (amygdala, hypothalamus) BV-2 microglial cells N9 microglial cells forced swim test in mice (hippocampal tissue) LPS-injected mice Aβ1-42-induced dementia in rats transient middle cerebral artery occlusion in rats rats with induced ischemic strokeBV-2 microglial cells LPS-injected mice BV-2 cells streptozotocin-induced dementia in rats pilocarpine-induced status epilepticus in mice AlCl_3_-induced brain damage in rats (hippocamups, cortex) thioacetamice-induced hepatic encephalopathy in mice	[53] [103] [54] [55] [51] [62] [61] [63] [65] [75] [76] [85] [80] [81] [90] [91] [89] [94]
↓IL-1β	schisandrin B schisandrol B (gomisin A) *Schisandra* lignans *Schisandra* essential oil *E. senticosus* extract *R. rosea* extract, rosin, rosarin, salidroside withanone withanolide A *W. somnifera* extract	micorglia-neuron cocultures BV-2 microglial cells forced swim test (amygdala, hypothalamus) subarachnoid hemorrhage in rats N9 microglial cells BV-2 microglial cells forced swim test in mice (hippocamp tissue) Aβ1-42-induced dementia in rats rats with induced ischemic stroke BV-2 microglial cells streptozotocin-induced dementia in rats pilocarpine-induced status epilepticus in mice transgenic mice with ALS	[103] [54] [55] [57] [62] [61] [65] [76] [80] [90] [91] [101]
↓IL-6	schisandrin A schisandrin B schisandrin C schisandrol B (gomisin A) *Schisandra* lignans *E. senticosus* extract salidroside *R. rosea* extract, rosin, rosarin, salidroside *R. rosea* extract *R. rosea* extract (standardized for salidroside) withanone *W. somnifera* extract	BV-2 microglial cells, mouse primary microglia micorglia–neuron cocultures BV-2 microglial cells forced swim test (amygdala, hypothalamus) BV-2 microglial cells N9 microglial cells forced swim test in mice (hippocampal tissue) rats with induced ischemic stroke transient middle cerebral artery occlusion in rats BV-2 microglial cells BV-2 cells autoimmune encephalomyelitis in mice streptozotocin-induced dementia in rats transgenic mice with ALS	[53] [103] [54] [55] [51] [62] [61] [76] [85] [80] [81] [82] [90] [101]
↓IFN-γ	*R. rosea* extract (standardized for salidroside) withanone *W. somnifera* extract	autoimmune encephalomyelitis in mice streptozotocin-induced dementia in rats transgenic mice with ALS	[82] [90] [101]
↓iNOS	schisandrin B schisandrin C *Schisandra* lignans *R. rosea* extract, rosin, rosarin, salidroside *E. senticosus* extract	BV-2 microglial cells forced swim test (amygdala, hypothalamus) BV-2 microglial cells forced swim test in mice (hippocampal tissue) BV-2 microglial cells rats with induced ischemic stroke	[54] [55] [51] [61] [80] [75]
↓NO	schisandrin A schisandrin B withanolide A withaferin A *W. somnifera* extract	BV-2 microglial cells, mouse primary microglia micorglia–neuron cocultures BV-2 microglial cells BV-2 microglial cells BV-2 microglial cells	[53] [103] [98] [98] [98]
↓COX-2	schisandrin B schisandrin C *Schisandra* lignans *E. senticosus* extract	BV-2 microglial cells forced swim test (amygdala, hypothalamus) BV-2 microglial cells forced swim test in mice (hippocamp) rats with induced global cerebral ischemia rats with induced ischemic stroke	[54] [55] [51] [61] [74] [75]
↓PGE_2_	schisandrin B schisandrin C	micorglia–neuron cocultures BV-2 microglial cells BV-2 microglial cells	[103] [54] [51]
↓NF-κB	schisandrin A schisandrin B schisandrin C schisandrol B (gomisin A) SCP-2 salidroside *R. rosea* extract withanolide A withaferin A *W. somnifera* extract	BV-2 microglial cells, mouse primary microglia micorglia–neuron cocultures BV-2 microglial cells middle cerebral artery occlusion and reperfusion in rats subarachnoid hemorrhage in rats BV-2 microglial cells N9 microglial cells LPS-injected mice transient middle cerebral artery occlusion in rats LPS-injected rats BV-2 cells AD in mice transgenic mice with FTLD thioacetamice-induced hepatic encephalopathy LPS-injected rats transgenic mice with ALS	[53] [103] [54] [56] [57] [51] [62] [63] [85] [86] [81] [92] [101] [94] [99] [101]
↓MAPK	schisandrin C SCP-2 *R. rosea* extract *R. rosea* extract *W. somnifera* extract	BV-2 microglial cells LPS-injected mice LPS-injected mice BV-2 cells thioacetamice-induced hepatic encephalopathy LPS-injected rats	[51] [63] [80] [81] [94] [99]
↓JAK/STAT activation	schisandrin A schisandrin C *Schisandra* lignans micrandilactone C *R. rosea* extract (standardized for salidroside)	BV-2 microglial cells, mouse primary microglia BV-2 microglial cells forced swim test in mice (hippocampal tissue) 3-NPA-induced HD in mice autoimmune encephalomyelitis in mice	[53] [51] [61] [66] [82]
↓inflammasome formation	schisandrin B withanolide A	subarachnoid hemorrhage in rats AD in mice	[57] [92]
↓MCP-1	withanone *W. somnifera* extract	streptozotocin-induced dementia in rats transgenic mice with ALS	[90] [101]
↑Nrf-2	schisandrin C salidroside withanolide A withaferin A *W. somnifera* extract	BV-2 microglial cells transient middle cerebral artery occlusion in rats LPS-injected rats BV-2 microglial cells BV-2 microglial cells thioacetamice-induced hepatic encephalopathy BV-2 microglial cells	[51] [85] [86] [98 [98] [94] [98]
↑HO-1	salidroside withanolide A withaferin A *W. somnifera* extract	transient middle cerebral artery occlusion in rats LPS-injected rats BV-2 microglial cells BV-2 microglial cells thioacetamice-induced hepatic encephalopathy BV-2 microglial cells	[85] [87] [98] [98] [94] [98]
↑IL-4	*E. senticosus* extract *R. rosea* extract (standardized for salidroside) *W. somnifera* extract	rats with induced ischemic stroke autoimmune encephalomyelitis in mice transgenic mice with ALS	[75] [82] [101]
↑IL-10	*E. senticosus* extract	rats with induced ischemic stroke	[75]

↑ increase, ↓ decrease; *S. chinensis*—*Schisandra chinensis*; *E. senticosus*—*Eleuterococcus senticosus*; *R. rosea*—*Rhodiola rosea*; *W. somnifera*—*Withania somnifera*; 3-NPA—3-nitropropionic acid; AD—Alzheimer’s disease; COX-2—cyclooxygenase-2; HO-1—hemoxygenase-1; IL—interleukin; IFN-γ—interferon-γ; iNOS—inducible NO synthase; JAK/STAT—prostaglandin E_2_; MAPK—mitogen-activated protein kinase; MCP-1—monocyte chemoattractant protein-1; NF-κB—nuclear factor kappa-light-chain-enhancer of activated B cells; NO—nitrogen oxide; Nrf-2—nuclear factor erythroid 2-related factor 2; PGE_2_—prostaglandin E_2_; PI3K/Akt—phosphatidylinositol-3 kinase/protein kinase B; SCP—Schisandra crude polysaccharide; SCP-2—purified fraction of SCP; TNF-α—tumor necrosis factor α.

## Data Availability

Not applicable.

## Abbreviations

AD	Alzheimer’s disease
AKT	protein kinase B (PKB)
AP-1	activator protein-1
ATP	adenosine triphosphate
BDNF	brain-derived neurotrophic factor
COX	cyclooxygenase
CNS	central nervous system
ERK	extracellular signal-regulated kinase
GDNF	glial-derived neurotrophic factor
GFAP	glial fibrillary acidic protein
GSK-3	glycogen synthase kinase-3
HD	Huntington’s disease
HO-1	hemoxygenase-1
IFN-γ	interferon-γ
IL	interleukin
iNOS	inducible NO synthase
JAK/STAT	janus kinase/signal transducer and activator of transcription
JNK	c-Jun N-terminal kinase
LPS	lipopolysaccharide
MAPK	mitogen-activated protein kinase
M2	anti-inflammatory microglial phenotype
MCP-1	monocyte chemoattractant protein-1
MS	multiple sclerosis
NF-κB	nuclear factor kappa-light-chain-enhancer of activated B cells
NLRP	nucleotide-binding oligomerization domain, leucine-rich repeat, and pyrin domain,
NO	nitric oxide
NOS	NO synthase
NPY	neuropeptide Y
Nrf-2	nuclear factor erythroid 2-related factor 2
PGE_2_	prostaglandin E_2_
PI3K/AKT	phosphatidylinositol-3 kinase/protein kinase B
PD	Parkinson’s disease
PKB	protein kinase B (AKT)
PPAR-γ	proliferator-activated receptor γ
PRR	pattern recognition receptor
TGF-β	transforming growth factor β
TLR	toll-like receptor
TNF-α	tumor necrosis factor-α
TNFR	tumor necrosis factor receptor
TRADD	TNFR1 signal transducer
TRAF	TNFR-associated factor
VEGF	vascular endothelial growth factor
